# Supplementary material for: Deliberately ignoring inequality to avoid rejecting unfair offers
Source: Commun Psychol. 2024 May 24;2:48. doi: 10.1038/s44271-024-00093-6 (PMC11332100; doi:10.1038/s44271-024-00093-6)
Supplement: Supplementary file 1 — Supplementary Information [file 44271_2024_93_MOESM1_ESM.pdf]

Supplementary information

Supplementary Note 1: Formal predictions for the Ultimatum Game

Fehr and Schmidt (1999) propose a theory of fairness, competition, and cooperation with a set of n players indexed by $i \in \{1, \ldots, n\}$ receiving monetary payoffs $x = x_1, \ldots, x_n$. Their utility functions are given by

$$(1) \qquad U_i(x) = x_i - \alpha_i \frac{1}{n-1} \sum \max\{x_j - x_i, 0\} - \beta_i \frac{1}{n-1} \sum \max\{x_i - x_j, 0\}$$

with the assumption that $\beta_i \leq \alpha_i$ and $0 \leq \beta_i \leq 1$. In the model, α_i represents the players' aversion to disadvantageous inequality and β_i their aversion to advantageous inequality.

Fehr and Schmidt apply their model to the ultimatum game in which a proposer offers a share $s \in [0,1]$ to a responder. The preferences of proposers and responders are represented by (α_1, β_1) and (α_2, β_2), respectively, and the responder can either accept or reject the offer. If the offer is accepted, the payoff vector is $(x_1, x_2) = (1 - s, s)$. If the offer is rejected, the vector is $(x_1, x_2) = (0,0)$. The empirical prediction for rejections by responders is expressed in Proposition 1 where the dominant strategy is to accept any offer $s \geq 0.5$ and to reject s if

$$(2) \qquad s < s'(\alpha_2) \equiv \frac{\alpha_2}{1 + 2\alpha_2} < 0.5$$

That is, rejections in the UG depend on the responders' aversion to disadvantageous inequality and the size of the offered share. By making rejections in the UG dependent on inequality and preferences for inequity aversion, Fehr and Schmidt can explain costly punishment in UG.

Supplementary Note 2: First Pilot Study

The objective of our first pilot study was to detect the classic inequality effect as a manipulation check and to assess the appropriateness of our exclusion criteria and comprehension checks. We implemented the study design as for the main study, specified in our methods section. To determine the required sample size, we conducted a power analysis in four steps. First, we extracted earlier reported rejection rates from the literature, finding average rejection rates of 28% for moderate inequality (i.e., 70/30 splits) and 68% for high inequality (i.e., 90/10 splits). Second, we converted the expected percentage point difference for rejection rates under moderate and high inequality to a standardized effect size, resulting in an expected effect of Cohen's $d = 0.874$ for the pilot study. Third, we assumed this effect size together with $\alpha = \beta = 0.05$ in a power analysis for a two-sided, two-sample t-test with the pwr package in R, resulting in a sample size of 141 across all four treatments. Finally, we added a margin of 23 subjects to account for exclusions based on our predefined exclusion criteria, resulting in a target sample size of 164. We recruited 165 US participants via Prolific. The mean age of our participants was 38 years (SD = 13). Supplementary Table 1 displays demographic information on the participants from pilot study 1.

Variable	Frequency (n)	Proportion (%)
Gender		
Men	66	40
Women	94	57
Prefer not to say	5	3
Education		
High school degree	39	24
Associate's degree	21	13
Bachelor's degree	64	39
Master's degree or PhD	36	22
Other (e.g., trade certificate)	2	1
Prefer not to say	3	2
Ethnicity		
Caucasian or white	89	54
African or African American	20	12
Asian or Asian American	24	15
Hispanic, Latino, or Latina	20	12
Multiracial or Mixed	7	4
Prefer not to say	5	3

Supplementary Table 2.1: Demographic Information for pilot study one. The characteristics of our survey respondents are based on answers to the following question: 1) "What is your gender?" 2) "What is your highest level of education completed?" and 3) "Which of the following best describes your ethnicity?".

Based on our predefined criteria, we excluded 20 participants from our data analysis: 18 participants failed to answer the comprehension questions correctly and two participants played a UG with an endowment of 60 cents – a measure to avoid deception. Observations from the remaining 145 participants were used in a regression of punishment on inequality. The sample sizes for treatments one to four were 33, 37, 37, and 38, respectively. Inequality significantly predicted punishment (β = 0.38, t(143) = 5.055, p < 0.001, 95% CI [0.235, 0.525]). The prediction remained significant when including non-understanding participants. The rejection rate under moderate inequality was 17% (t(143) = 3.211, p < 0.001, 95% CI [0.066, 0.274]). The model explained a moderate proportion of variance in punishment (F(1,143)=25.55, Adj. R^2= 0.15, SE = 0.45, p < 0.001).

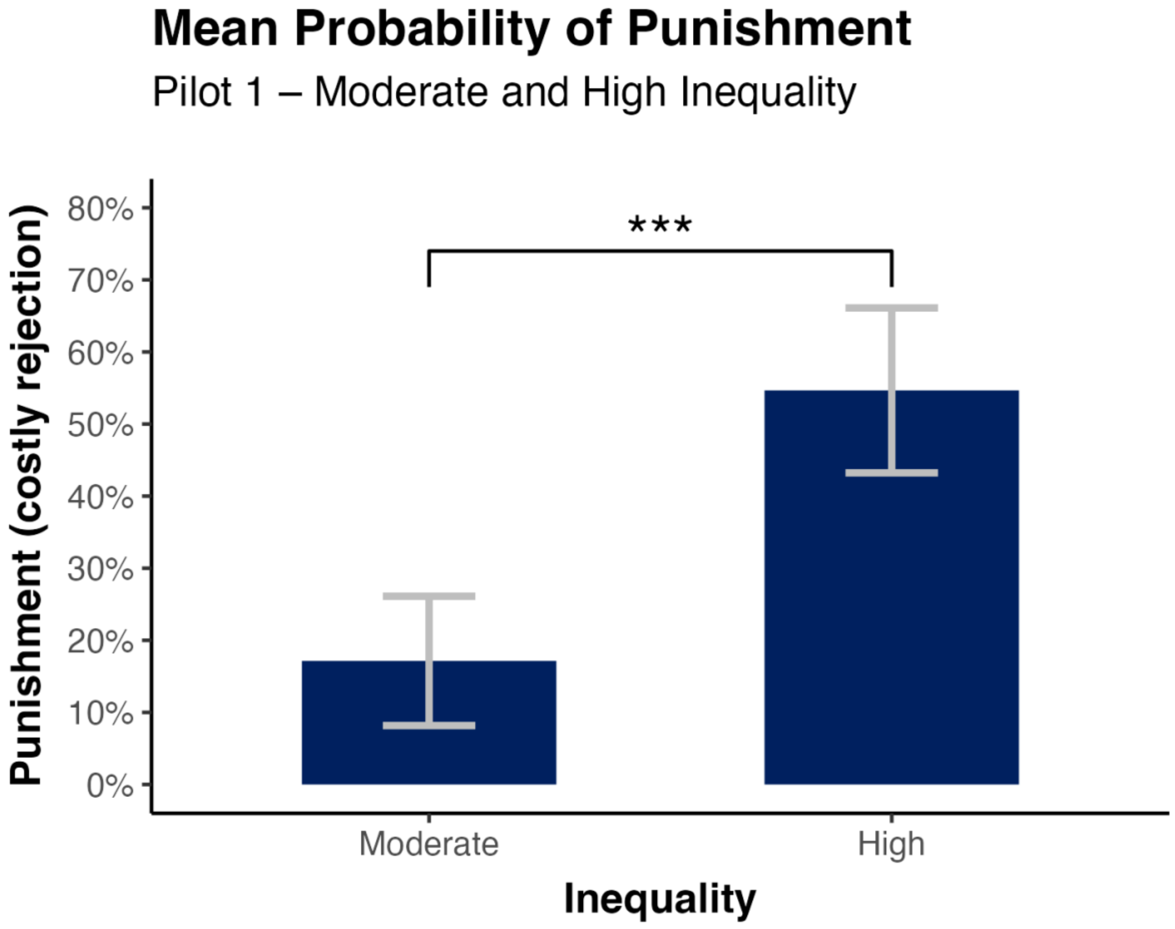

Supplementary Figure 2.1: Punishment in Pilot Study 1. The first pilot study provided evidence for a positive effect of inequality on punishment (*** p < 0.001). The mean rejection rates under moderate (n_M=70) and high (n_H=75) inequality are 17% and 55%, respectively. Error bars represent 95% confidence intervals.

The first pilot study revealed a ceiling effect in information search in that 95% of participants in moderate and high inequality treatments decided to seek information. We hypothesized that this finding resulted from deviations from study designs previously used in the literature. In particular, we had, contrary to earlier studies (e.g., Grossman, 2014), not informed participants in the first pilot about how the other person's endowment had been determined (randomly by a computer), whether the other person would be informed about information seeking or not (no information about ignorance is passed on), and whether the interaction would be anonymous or not (there is no possibility for follow-up interactions). Furthermore, we had asked participants in uncertainty treatments to confirm that they can seek free information (and excluded them if they did not answer this comprehension question correctly), which could have inadvertently generated demand for information. As an alternative explanation, we also saw the possibility that participants would only ignore information if information seeking would come at an additional cost. To examine these competing explanations, we conducted a second pilot study.

Supplementary Note 3: Second Pilot Study

The objective of the second pilot study was to seek additional information on participants' demand for information. In particular, the second pilot study was designed to examine whether ceiling effects in information search would still occur with study designs previously used in the literature (here: on the basis of Dana et al., 2007) or whether ceiling effects could only be addressed through an introduction of costs for seeking information. To assess these competing explanations, we conducted a second pilot with four conditions, all of which had moderate inequality and uncertainty.

The first condition was intended to serve as a control condition by replicating the ceiling effect from the first pilot study by using the same design. The second condition implemented a protocol by Grossman (2014) replicating the "hidden information" treatment by Dana et al. (2007). The third condition extended the second condition by additional costs of 10 cents for seeking information. The fourth condition introduced a cost of 20 cents. Treatments three and four combined were based on evidence that increases in costs of information decrease the demand for information (Serra-Garcia & Szech, 2022) – testing the alternative explanation that participants would only ignore information if information search would come at an extra cost. We recruited $n_2 = 164$ US participants via Prolific who had not participated in the first pilot study. The mean age of our participants was 39 years (SD = 15). Supplementary Table 2 displays demographic information on the participants from pilot study 2.

Variable	Frequency (n)	Proportion (%)
Gender		
Men	69	42
Women	92	56
Other (e.g., non-binary)	2	1
Prefer not to say	1	1
Education		
High school degree	53	32
Associate’s degree	20	12
Bachelor’s degree	59	36
Master’s degree or PhD	28	17
Other (some college)	3	2
Prefer not to say	1	1
Ethnicity		
Caucasian or white	98	60
African or African American	18	11
Asian or Asian American	22	13
Hispanic, Latino, or Latina	17	10
Multiracial or Mixed	5	3
Other (e.g., Middle Eastern)	4	2

Supplementary Table 3.1: Demographic Information for pilot study two. The characteristics of our survey respondents are based on answers to the following question: 1) “What is your gender?” 2) “What is your highest level of education completed?” and 3) “Which of the following best describes your ethnicity?”.

Based on our predefined criteria, we excluded 26 participants from our data analysis: 17 participants failed to answer all three comprehension questions correctly within two attempts, eight participants played a UG with an endowment of 60 cents to avoid deception, and one participant stated that their data should not be included in the data analysis due to non-seriousness in participation. Observations from the remaining 138 participants were used to compare ignorance rates across conditions. The sample sizes for conditions one to four were 36, 39, 37, and 26, respectively. For conditions 1, 2, 3, and 4, we observed ignorance rates of 25%, 64%, 95%, and 100%, respectively. These findings suggest that an introduction of additional costs for seeking information can be expected to lead to a ceiling effect in ignorance, and that a protocol based on the Grossman (2014) operationalization of the “hidden information” treatment by Dana and colleagues (2007) can be expected to neither lead to a floor nor a ceiling effect in ignorance.

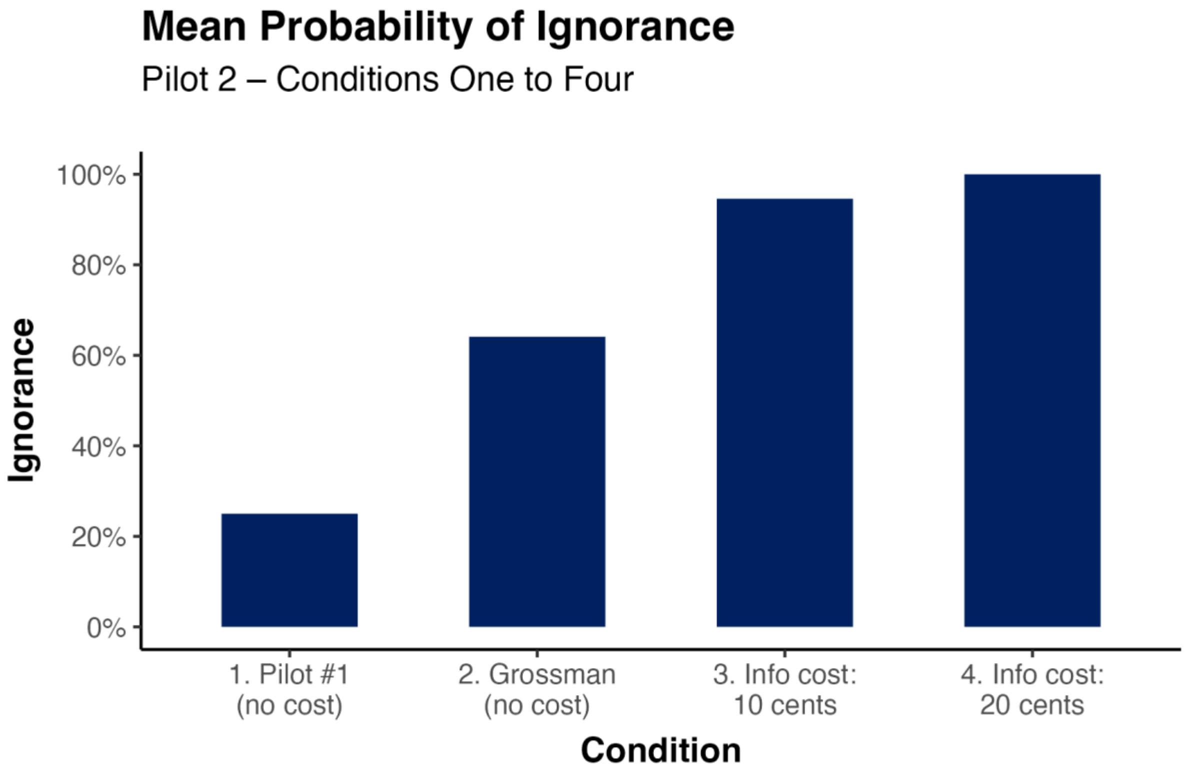

Supplementary Figure 3.1: Ignorance in Pilot Study 2. The second pilot study provided information on participants' demand for information. Condition one uses the design from pilot 1. Condition two is based on Dana et al. (2007) as described in Grossman (2014). Conditions three and four introduce additional costs for seeking information of 10 cents and 20 cents, respectively, to the design implemented in condition two. The mean probabilities of ignorance for conditions one through four are 25%, 64%, 95%, and 100%.

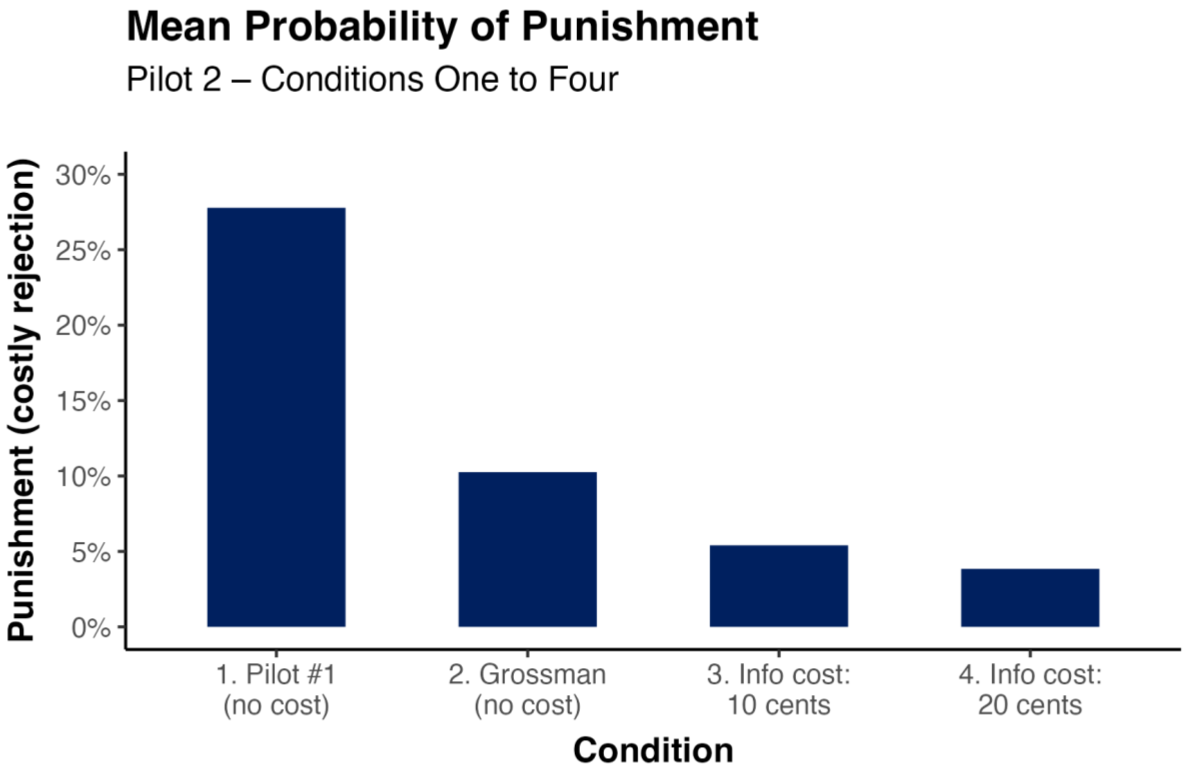

Supplementary Figure 3.2: Punishment in Pilot Study 2. The level of inequality was moderate for all conditions in the second pilot, where we observed mean probabilities of punishment of 28%, 10%, 5%, and 4% for conditions one to four, respectively. These findings are in line with our expectations for research question three in that punishment decreases (Supplementary Figure 3) as ignorance increases (Supplementary Figure 2).

Supplementary Note 4: Robustness Analysis

Of our 1,430 recruited participants, 99 did not fulfil our predefined inclusion criteria: 53 participants played a UG with an endowment of 60 or 20 cents to avoid deception, 7 participants stated that their data should not be included in our data analysis due to non-seriousness in participation, and 39 participants did not answer all three comprehension questions correctly within two attempts.

Since our results did not differ depending on whether or not we excluded non-understanding participants, we report results with non-understanding participants in our article—in line with our preregistered data analysis plan. As a robustness check, we report results without non-understanding participants in this part of the supplementary information (N_{SI} = 1,331):

- RQ1: The rejection rates under certainty and uncertainty were 18.3% and 21.2%, respectively. This difference was not significant in our model (β_{11} = 0.03, t(1329) = 1.33, p = 0.185, 95% CI [-0.013, 0.073]). Hence, we could not reject H10 when excluding non-understanding participants.
- RQ2: Excluding non-understanding participants, we also did not find a significant interaction between uncertainty and inequality (β_{23} = -0.04, t(1327) = -0.845, p = 0.398, 95% CI [-0.124, 0.044]).
- RQ3: In line with our expectation (H3A), we found that ignorance significantly predicted punishment when excluding non-understanding participants (β_{31} = -0.33, t(678) = -11.53, p < 0.001, 95% CI [-0.387, -0.273]). The overall fit of the regression model was significant (F(1, 678) = 132.9, R^2 = 0.164, SE = 0.37, p < 0.001).

Our robustness analyses for the three RQs show that the results do not change when excluding non-understanding participants. All analyses with and without non-understanding participants can be replicated with our data analysis pipeline on the OSF (see code availability statement).

Supplementary Note 5: Exploratory Analysis

Our first additional analysis compares the mean probability of punishment between deliberately ignorant and directly informed responders. For this comparison, we conducted a bivariate linear regression predicting the probability of punishment (y) by a dummy variable for the first and second informational state (s_{d1}), taking the value zero for deliberately ignorant and one for directly informed responders, with individuals $i = 1, \ldots, n$, and ε_i as the error term:

$$y_i = \beta_{50} + \beta_{51}\, s_{d1_i} + \varepsilon_i \tag{3}$$

We found that the dummy variable for the informational state significantly predicted punishment (β_{51} = 0.129, t(1037) = 5.796, p < 0.001, 95% CI [0.086, 0.172]), based on a mean probability of punishment of 5.7% for deliberately ignorant responders (t(1037) = 3.202, p = 0.001, 95% CI [0.022, 0.092]).

Our second additional analysis compared the mean probability of punishment by directly informed and information-seeking responders. For this comparison, we conducted a second bivariate linear regression predicting the probability of punishment (y) by a second dummy variable for the first and third informational state (s_{d2}), taking the value zero for directly informed and one for information-seeking responders, with individuals $i = 1, \ldots, n$, and ε_i as the error term:

$$y_i = \beta_{60} + \beta_{61}\, s_{d2_i} + \varepsilon_i \tag{4}$$

As in our first additional analysis, we found that the informational state significantly predicted punishment (β_{61} = 0.201, t(1001) = 7.043, p < 0.001, 95% CI [0.144, 0.258]). The mean probability of punishment for directly informed responders was 18.6% (t(1001) = 11.363, p < 0.001, 95% CI [0.155, 0.217]).
