# Supplementary material for: Deliberately ignoring inequality to avoid rejecting unfair offers
Source: Commun Psychol. 2024 May 24;2:48. doi: 10.1038/s44271-024-00093-6 (PMC11332100; doi:10.1038/s44271-024-00093-6)
Supplement: Supplementary file 3 — Reporting Summary [file 44271_2024_93_MOESM3_ESM.pdf]

nature portfolio

Corresponding author(s): Konstantin Offer

Last updated by author(s): Mar 20, 2024

Reporting Summary

Nature Portfolio wishes to improve the reproducibility of the work that we publish. This form provides structure for consistency and transparency in reporting. For further information on Nature Portfolio policies, see our Editorial Policies and the Editorial Policy Checklist.

Statistics

For all statistical analyses, confirm that the following items are present in the figure legend, table legend, main text, or Methods section.

n/a	Confirmed	
☐	☒	The exact sample size (*n*) for each experimental group/condition, given as a discrete number and unit of measurement
☐	☒	A statement on whether measurements were taken from distinct samples or whether the same sample was measured repeatedly
☐	☒	The statistical test(s) used AND whether they are one- or two-sided<br>*Only common tests should be described solely by name; describe more complex techniques in the Methods section.*
☐	☒	A description of all covariates tested
☐	☒	A description of any assumptions or corrections, such as tests of normality and adjustment for multiple comparisons
☐	☒	A full description of the statistical parameters including central tendency (e.g. means) or other basic estimates (e.g. regression coefficient) AND variation (e.g. standard deviation) or associated estimates of uncertainty (e.g. confidence intervals)
☐	☒	For null hypothesis testing, the test statistic (e.g. *F*, *t*, *r*) with confidence intervals, effect sizes, degrees of freedom and *P* value noted<br>*Give P values as exact values whenever suitable.*
☒	☐	For Bayesian analysis, information on the choice of priors and Markov chain Monte Carlo settings
☒	☐	For hierarchical and complex designs, identification of the appropriate level for tests and full reporting of outcomes
☐	☒	Estimates of effect sizes (e.g. Cohen's *d*, Pearson's *r*), indicating how they were calculated

Our web collection on statistics for biologists contains articles on many of the points above.

Software and code

Policy information about availability of computer code

Data collection	All data has been collected in a Qualtrics-based online experiment and all analyses pipelines, power analyses scripts, and simulated data analyses code for the Stage 2 manuscript have been made available at the OSF (see link in data availability statement).
Data analysis	All analyses pipelines, power analyses scripts, and simulated data analyses code for the Stage 2 manuscript have been made available at the OSF (see link in data availability statement). All statistical analyses were performed using the R programming language, and regression models were built with the R “stats” package (version 4.3.1).

For manuscripts utilizing custom algorithms or software that are central to the research but not yet described in published literature, software must be made available to editors and reviewers. We strongly encourage code deposition in a community repository (e.g. GitHub). See the Nature Portfolio guidelines for submitting code & software for further information.

Data

Policy information about availability of data

All manuscripts must include a data availability statement. This statement should provide the following information, where applicable:

- Accession codes, unique identifiers, or web links for publicly available datasets
- A description of any restrictions on data availability
- For clinical datasets or third party data, please ensure that the statement adheres to our policy

All anonymized raw data and materials have been made available for the Stage 2 manuscript at the OSF (see link in data availability statement).

March 2021

Human research participants

Policy information about studies involving human research participants and Sex and Gender in Research.

Reporting on sex and gender	For pilot study 1, we recruited 165 US participants via Prolific. Of these participants, 40% identified as men, 57% as women, and 3% as other. For pilot study 2, we recruited 164 US participants via Prolific. Of these participants, 42% identified as men, 56% as women, 1% identified as other, and 1% preferred not to say. For the main study, we recruited 1,430 US participants via Prolific. Of these participants, 51% identified as men, 47% as women, 1% as other, and 1% preferred not to say. Sex- or gender-based analyses were not conducted for studies since we did not have any tangible sex- or gender-based scientific hypotheses.
Population characteristics	See "behavioural & social sciences study design" section.
Recruitment	All participants were recruited via Prolific. The Prolific sample was a non-representative US sample and all participants completed an informed consent form. Participant compensation was above 8$ per hour, in line with Prolific's pricing policy.
Ethics oversight	The study was approved under the ethical regulations of the Max Planck Institute for Research on Collective Goods in Bonn, Germany.

Note that full information on the approval of the study protocol must also be provided in the manuscript.

Field-specific reporting

Please select the one below that is the best fit for your research. If you are not sure, read the appropriate sections before making your selection.

☐ Life sciences ☒ Behavioural & social sciences ☐ Ecological, evolutionary & environmental sciences

For a reference copy of the document with all sections, see nature.com/documents/nr-reporting-summary-flat.pdf

Behavioural & social sciences study design

All studies must disclose on these points even when the disclosure is negative.

Study description	Quantitative experiment with 2x2 factorial design implemented in online survey.
Research sample	For pilot study 1, we recruited a non-representative sample of 165 US participants via Prolific. The mean age of our participants was 38 years (SD = 13). The highest level of education completed was a high school or associate's degree for 36% of participants, a bachelor's degree for 39% of participants, a master's degree or doctorate degree for 22% of participants, while 2% of participants had a different educational attainment (some college / trade certificate), and 1% preferred not to say. The majority of participants identified as Caucasian or white (54%), 12% identified as African or African American, 15% as Asian or Asian American, 12% as Hispanic, Latino, or Latina, 4% as multiracial or mixed, and 3% of participants preferred not to state their ethnic identification.<br><br>For pilot study 2, we recruited a non-representative sample of 164 US participants via Prolific. The mean age of our participants was 39 years (SD = 15). The highest level of education completed was high school for 32% of participants, an associate's degree for 12% of participants, a bachelor's degree for 36% of participants, a master's degree or doctorate degree for 17% of participants, while 1% of participants had a different educational attainment (some college), and 2% preferred not to say. The majority of participants identified as Caucasian or white (59.8%), 11% identified as African or African American, 13.4% as Asian or Asian American, 10.4% as Hispanic, Latino, or Latina, 3% as multiracial or mixed, and 2.4% of participants preferred not to state their ethnic identification.<br><br>For the main study, we recruited a non-representative sample of 1,430 US participants via Prolific. The mean age of our participants was 41 years (SD = 13). The highest level of education completed was a high school degree for 27% of participants, an associate's degree for 13% of participants, a bachelor's degree for 41% of participants, a master's degree or doctorate degree for 16% of participants, while 2% of participants had a different educational attainment (e.g., some college), and 1% preferred not to say. The majority of participants identified as Caucasian or white (66%), 11% identified as African or African American, 11% as Asian or Asian American, 6% as Hispanic, Latino, or Latina, 1% as Native American or Indigenous, 4% as multiracial or mixed, and 1% of participants preferred not to state their ethnic identification.
Sampling strategy	The sample size for the main study is based on three a priori power analyses as described in the methods section of the Stage 1 manuscript. The sample sizes for the pilot studies is based on one a priori power analysis as described in the Supplementary Information Section 2. All subjects were randomly assigned to experimental treatments.
Data collection	Data for pilot studies 1 and 2 and the main study was collected in a Qualtrics-based online experiment, as described in the methods section of the Stage 1 manuscript and in the Supplementary Information file.
Timing	Data for pilot study 1 was collected on 26/07/2023.<br>Data for pilot study 2 was collected on 08/08/2023.<br>Data for the main study was collected on 15/11/2023.

Data exclusions	Based on our predefined criteria, we excluded 20 participants from our data analysis for pilot study 1. In particular, 18 participants failed to answer all three comprehension questions correctly within two attempts, and two participants played an ultimatum game with an endowment of 60 cents to avoid deception.<br><br>Based on our predefined criteria, we excluded 26 participants from our data analysis for pilot study 2. In particular, 17 participants failed to answer all three comprehension questions correctly within two attempts, eight participants played an ultimatum game with an endowment of 60 cents to avoid deception, and one participant stated that their data should not be included in the data analysis due to non-seriousness in participation.<br><br>In the main study, 99 of our recruited participants did not fulfil the three predefined inclusion criteria: 53 participants played a UG with an endowment of 60 or 20 cents to avoid deception, 7 participants stated that their data should not be included in the data analysis due to non-seriousness in participation, and 39 participants failed to answer all three comprehension questions correctly within two attempts. In line with our preregistration, we performed all of our analyses for research questions one to three with and without non-understanding participants. The results did not differ when including non-understanding participants. Consequently, we only excluded 60 participants from our data analysis. We report observations for the remaining 1370 participants in the article.
Non-participation	No participant who provided informed consent dropped out of pilot studies 1 and 2 and the main study.
Randomization	Participants were randomly assigned to the experimental treatments in pilot studies 1 and 2 and in the main study, as described in the methods section of the Stage 1 manuscript.

Reporting for specific materials, systems and methods

We require information from authors about some types of materials, experimental systems and methods used in many studies. Here, indicate whether each material, system or method listed is relevant to your study. If you are not sure if a list item applies to your research, read the appropriate section before selecting a response.

Materials & experimental systems

n/a	Involved in the study
☒	☐ Antibodies
☒	☐ Eukaryotic cell lines
☒	☐ Palaeontology and archaeology
☒	☐ Animals and other organisms
☒	☐ Clinical data
☒	☐ Dual use research of concern

Methods

n/a	Involved in the study
☒	☐ ChIP-seq
☒	☐ Flow cytometry
☒	☐ MRI-based neuroimaging
